# Metformin as a Potential Agent in the Treatment of Multiple Sclerosis

**DOI:** 10.3390/ijms21175957

**Published:** 2020-08-19

**Authors:** Angela Dziedzic, Joanna Saluk-Bijak, Elzbieta Miller, Michal Bijak

**Affiliations:** 1 Department of General Biochemistry, Faculty of Biology and Environmental Protection, University of Lodz, Pomorska 141/143, 90-236 Lodz, Poland; angela.dziedzic@unilodz.eu (A.D.); joanna.saluk@biol.uni.lodz.pl (J.S.-B.); 2 Department of Neurological Rehabilitation, Medical University of Lodz, Milionowa 14, 93-113 Lodz, Poland; elzbieta.dorota.miller@umed.lodz.pl; 3 Biohazard Prevention Centre, Faculty of Biology and Environmental Protection, University of Lodz, Pomorska 141/143, 90-236 Lodz, Poland

**Keywords:** metformin, neurodegeneration, multiple sclerosis

## Abstract

Metformin, a synthetic derivative of guanidine, is commonly used as an oral antidiabetic agent and is considered a multi-vector application agent in the treatment of other inflammatory diseases. Recent studies have confirmed the beneficial effect of metformin on immune cells, with special emphasis on immunological mechanisms. Multiple Sclerosis (MS) is an autoimmune disease of the central nervous system (CNS) characterized by various clinical courses. Although the pathophysiology of MS remains unknown, it is most likely a combination of disturbances of the immune system and biochemical pathways with a disruption of blood–brain barrier (BBB), and it is strictly related to injury of intracerebral blood vessels. Metformin has properties which are greatly desirable for MS therapy, including antioxidant, anti-inflammatory or antiplatelet functions. The latest reports relating to the cardiovascular disease confirm an increased risk of ischemic events in MS patients, which are directly associated with a coagulation cascade and an elevated pro-thrombotic platelet function. Hence, this review examines the potential favourable effects of metformin in the course of MS, its role in preventing inflammation and endothelial dysfunction, as well as its potential antiplatelet role.

## 1. Characteristics of Metformin

Metformin (1,1-dimethylbiguanide hydrochloride) is a synthetic derivative of guanidine isolated from the extract of the French lilac (*Galega officinalis*) [1], which possess hypoglycemic activity [2]. Metformin is the first-line drug in the treatment of type 2 diabetes (T2D), especially in patients with obesity [3]. It is easily soluble in water and nearly insoluble in organic compounds, such as acetone, ether or chloroform [4]. Metformin is a crystalline, hygroscopic white powder with a rather bitter taste and a half-life of between 2–6 h [5]. During controlled clinical trials, maximum metformin levels in human plasma do not exceed 5 µg/mL (38.8 µmol/L) [6]. The usual therapeutic oral doses of metformin range from 500 up to 3000 mg/day depending on the type of treated disease [7]. Metformin is absorbed incompletely (approximately 60%), predominantly from the small intestine. Subsequently, the remaining components pass to the colon, where it is excreted in faeces [8].

Several experimental and clinical studies reveal that metformin may have beneficial applications in the treatment of cancer [9,10,11] and polycystic ovary syndromes (PCOs) [12,13,14,15] and exhibits oligo- [16] and cardioprotective [17] properties. Metformin works in a multidirectional way, and besides its role in the treatment of diabetes, it mediates in many vital processes through various biochemical pathways. Metformin has anti-inflammatory [18,19,20] and antioxidant [21,22,23] features as well as the capacity to restore endothelial dysfunctions [24,25]. Furthermore, metformin exhibits a platelet anti-aggregation effect [26,27], which diminishes the formation of advanced glycation end-products [28,29] and predominantly explains the blood vascular protective effect.

In order to provide a broader understanding of metformin usage in humans, its side effects should be considered. The most dangerous effect associated with metformin administration is the occurrence of lactic acidosis. It was observed in about 3 cases per 1,000,000 patients after a long-term treatment [30]. Most often, side effects resulted from improper drug usage (too high dose) or the presence of comorbidities [31]. Gastrointestinal adverse effects (diarrhoea) occurred in less than 20% of patients and remitted when the dosage was reduced [32]. Despite many reports concerning the risk of usage of oral anti-diabetes drugs such as metformin, serious adverse events are predictable and potentially preventable if the prescribing guidelines are respected [33,34].

The way metformin works is omnidirectional but still not fully understood. The main function of metformin is based on sensitizing cells to insulin and on lowering the serum glucose level as well as on inhibiting the mitochondrial glycerol-3-phosphate dehydrogenase (GPDH) in the liver to suppress the process of gluconeogenesis and intensification of anaerobic glycolysis [35]. Furthermore, metformin activates the enzyme adenosine monophosphate kinase (AMPK), leading to inhibition of crucial enzymes engaged in glycogen synthesis in the liver, simultaneously stimulating insulin signalling and transport of glucose molecules to the muscles cells [36]. The above described effects may represent a strong contribution to the anti-inflammatory features of metformin. In this article, we will focus on a survey of the accessible reports regarding the effect of metformin on the immune and haemostasis system with an emphasis on immunological mechanisms associated with the development and preservation of autoimmunity and its virtual suitability in the treatment of autoimmune diseases.

## 2. Pathophysiology of MS

Multiple sclerosis (MS) is a chronic demyelinating autoimmune disease of the central nervous system (CNS) affecting over 2.5 million people worldwide [37]. In 1996, as a result of consensus, the four clinical courses of MS were defined: relapsing-remitting MS (RRMS), secondary-progressive MS (SPMS), primary-progressive MS (PPMS) and progressive-relapsing MS (PRMS) [38]. Further research concerning accurate clinical course descriptions (phenotypes) included clinically isolated syndrome (CIS) as a clinical descriptor RRMS, where the disease can be modified by targeting inflammation process and progressive MS (P-MS) identified by an accrual of gradual disability independent of relapses over time. CIS is recognized as the first demonstration of a clinical disease typical to MS, with no dissemination of lesions. RRMS is the most common MS form (55%), with differentiation in frequency and intensity of relapses. This MS phenotype can be inactive, due to immunomodulatory therapies, or active, during clinical intensification. P-MS can be distinguished into four subtypes for both the primary and secondary progressive types: active and inactive with progression, and active and inactive without progression [39,40]. Studies have proposed that genetic, environmental and infectious agents may be among the agents affecting the development of MS. Nevertheless, inflammation of the CNS is the main cause of damage in MS. Numerous immunological studies were carried out using experimental encephalomyelitis (EAE) as an animal model of autoimmune inflammatory diseases of CNS resembling MS [41,42,43]. Although EAE is not a perfect MS model, it allows a better understanding of the roles of particular immunological pathways potentially involved in a MS course. Furthermore, the EAE model was crucial in the development of Food and Drug Administration (FDA)-approved MS therapy [44].

The innate and adaptive immune responses are the leading players in the development of MS. The innate system retrieves an essential role in the initiation and progression of MS, affecting the function of T and B cells. Numerous studies have shown that excessive activity of myelin-specific T helper 17 (Th17) cells together with dysfunction of regulatory T cells (Tregs) play an important role in MS/EAE pathogenesis [45,46,47,48,49]. Th17 cells secrete a distinctive profile of pro-inflammatory cytokines, including interleukin (IL)-17A, IL-17F, IL-21 and tumour necrosis factor α (TNF-α) [50]. The secretion of IL-17 from infiltrating T cells into the CNS is associated with blood–brain barrier (BBB) disruption [51]. It is well documented that genetic deficiency of IL-17 or neutralization of IL-17 in mice with EAE leads to amelioration of clinical disease symptoms [52,53]. Th17 can also secrete anti-inflammatory cytokines, such as IL-10, IL-22 and IL-26, which may perform an essential protective function by reducing inflammation and tissue impairment [54,55]. In fact, Th17 cell-derived IL-10 has been found to have a pivotal role in the inhibition of Th17-driven inflammation in EAE [56]. Tregs act as negative regulators of inflammation and are distinguished by the expression of the transcription factor forkhead box P3 (FoxP3) [57]. It was registered that a disturbance in the function and amount of the Treg cell subset is related to the severity of the RRMS course [58]. FoxP3+ Tregs play an essential role in protection and recovery from EAE/MS by inhibiting autoreactive T cells, and either the lack of Tregs or their abnormal functions can potentiate the severity of EAE [59,60]. Therefore, the Th17/Treg balance as well as the participation of pro- and anti-inflammatory cytokines produced by them provide a foundation for better understanding of the molecular mechanisms of autoimmune regulation [61].

## 3. Overview of the Favourable Cellular Effects of Using Metformin

Metformin has a therapeutic effect, confirmed in an animal model of systemic lupus erythematosus (SLE) through mechanisms involving the inhibition of oxidative phosphorylation (OXPHOS) and the diminishment of CD4+ T cell activation. The activation of the signalling kinase AMPK is one of the most extensively studied metformin mechanisms. Metformin blocks complex I of the electron transport chain, thus inhibiting OXPHOS and adenosine triphosphate (ATP) production [62], leading to AMPK activation in numerous cell types [63]. There is evidence that mitochondrial dysfunction in oligodendrocytes and neurons contributes to MS/EAE pathomechanisms [64,65,66].

Accumulated evidence indicates that deregulated sphingolipid metabolism is related to a number of neurodegenerative diseases [67,68]. A study on metabolic changes in active MS revealed a shift in the lipid composition of white and grey matter to a higher phospholipid and lower sphingolipid content [69]. Ceramide is a long-chain fatty acid amide derivative of sphingosine which plays an important role in signal transduction in apoptosis [70]. Furthermore, ceramides may induce mitochondrial dysfunction, may enhance expression of pro-oxidative genes and glutamate excitotoxicity, as well as may diminish neuroprotective genes expression [71]. Vidaurre et al. found elevated levels of ceramide C16:0 and C24:0 in MS patient cerebrospinal fluid (CSF) without any changes in pro-inflammatory cytokine levels [71]. Fatty acids may induce apoptosis in astrocytes by extending de novo ceramide synthesis [72]. The recently published study demonstrated that long-term stimulation of AMPK activator, such as 5-aminoimidazole-4-carboxamide ribonucleotide (AICAR), prevented the astrocyte apoptosis. AICAR blockage of ceramide accumulation leads to an inhibition of the Raf-1/extracellular signal-regulated kinase cascade, which mediates fatty acid-induced apoptosis. These data indicate that AICAR may protect cells from stimulus apoptosis induced by stress [73]. The preclinical studies demonstrated that the combination of lovastatin and AICAR in therapy improved neuroprotection against inflammatory mediators-induced dysfunction in mitochondria and peroxisomes in EAE model [74]. Furthermore, there is evidence that the supplementation of AICAR with statin combination treatment may provide greater efficacy for MS than statin alone [75]. Due to insufficient molecular and clinical studies of metformin properties in MS, its effectiveness cannot be unambiguously established. However, there are studies which demonstrate that both AICAR and metformin attenuate the course of EAE via regulation of immune responses and preservation of BBB permeability [76,77]. Analogous to AICAR, metformin as an AMPK activator is potentially able to protect neuronal cells from apoptosis caused by metabolism disruption and ceramide accumulation. Furthermore, a study demonstrates that AMPK signalling protects oligodendrocytes and restores proper functioning of the CNS in EAE via inhibition of cellular infiltration and T cell autoimmunity [78].

There are three subtypes of the peroxisome proliferator-activated receptor (PPAR) family (α, γ and β/δ), and synthetic ligands for PPARα (fibrates) and PPARγ (Thiazolidinediones, TZDs) are currently prescribed for the respective management of T2D due to their ability to ameliorate hyperglycaemia by improving glucose metabolism of insulin target tissues [79]. TZDs and metformin modulate the endothelial function and atherosclerotic plaque formation via separate pathways. Rendering their combination appears to be a promising factor of slowing down diabetic atherosclerosis [80,81] and of reducing macrovascular complications of diabetes [82,83]. One of the main differences in their metabolic actions is that metformin mainly reduces hepatic glucose release, while TZDs predominantly increases insulin-stimulated glucose disposal into skeletal muscle [84]. However, TZDs, like metformin, inhibit complex I of the respiratory chain [85,86]. TZDs are the synthetic ligand for peroxisome proliferator-activated receptor (PPAR)γ [87]. It was stressed that the therapeutic potential of PPARγ ligands in the brain lead to TZD testing in neurodegeneration diseases [88]. PPARγ agonists affect a variety of physiological responses and are thought to contribute to the origin of demyelinating disease. Furthermore, PPARγ agonists can increase the neuronal survival rate and can reduce neuronal damage [89]. It may help to reduce irreversible neuronal damage occurring in the severe stages of MS [90]. Importantly for MS, PPAR appears to control inflammation induced by CD4+ T cell infiltration, at least in in vitro studies [91]. One of the promising PPARγ agonists is CHS-131, which can cross the BBB, stimulates anti-inflammatory activity and is clinically effective in EAE. The double-blind, parallel-group 2-part study randomized on 227 RRMS patients have demonstrated that the treatment with CHS-131 was generally well-tolerated and that the 3 mg dose resulted in a significant decrease in the number of new lesions in comparison to placebo patients [92]. The PPARα agonists have an equally crucial role in regulating immune responses and in ameliorating EAE course [93,94,95]. Metformin acts via the AMPK-Sirtuin-1-PPARα pathway and upregulates the insulin-like growth factor (IGF)-binding protein 2 (IGFBP-2) expression, enhancing insulin sensitivity in the cells [96].

Furthermore, metformin reduced oxygen consumption, activation and interferon (IFN)-γ production in CD4+ T cells of lupus mice, from healthy controls and SLE patients in vitro, as well as in vivo in lupus mice [97]. The latest research conducted by Titov et al. has shown that metformin inhibits the transcription of IFN-stimulated genes in CD4+ T cells from SLE patients and healthy volunteers and that these effects were increased by using inhibitors of the electron transport chain respiratory complexes [98]. Accordingly, this indicates that mitochondrial respiration is required for IFN-stimulated gene expression in CD4+ T cells and signalizes the existence of a novel mechanism by which metformin may exert a therapeutic effect in autoimmune diseases [98]. Studies suggest that metformin has an impact on crucial immunopathological mechanisms engaged in systemic autoimmune diseases, such as the Th17/Tregs balance, autoantibodies production, macrophage polarization and cytokine synthesis [99]. Duan et al. have shown that metformin suppressed T cell proliferation and differentiation of Th17 cells while promoted the development of Tregs in vitro [100]. They have demonstrated that administration of metformin to mice alleviated autoimmune insulitis and substantially decreased the number of pro-inflammatory IFN-γ+ and IL-17+CD4+ T cells [100]. Nath et al. showed that metformin (20–100 mg/kg) successfully slowed down the progression of the disease, reduced infiltration of immune cells and decreased the expression of pro-inflammatory cytokines (IFN-γ, TNF-α, IL-1β, IL-6 and IL-17) in the CNS of EAE animals [101]. Furthermore, they have demonstrated that the CNS from metformin-treated EAE animals exhibited a significantly lesser expression of pro-inflammatory mediators, such as matrix metalloproteinase 9 (MMP-9) and chemokine RANTES/CCL5 (Regulated upon Activation, Normal T Cell Expressed and Presumably Secreted) [101], which play a critical role in the infiltration of immune cells during EAE disease progression [102,103].

Axons of the neuronal cells are covered by a myelin sheath, made by oligodendrocytes derived from specializing stem cells, known as oligodendrocyte progenitor cells (OPCs). OPCs extensively presented in the grey and white matter of the CNS constitute approximately 5–8% of the total pool of brain cells, being a great repair source of myelin. Essentially, the remyelination ability remains ineffective and seems to reduce with age in spite of the considerable number of OPCs present in demyelinated MS lesions [104,105]. Korean research has shown that metformin augmented the protein expressions of anti-inflammatory cytokines (IL-4 and IL-10) as well as diminished the production of nitric oxide (NO), prostaglandin E2 (PGE2) and pro-inflammatory cytokines (IL-1β, IL-6 and TNF-α) by downregulation of nuclear factor (NF)-κB translocation in macrophages [106]. Furthermore, the study demonstrated that metformin inhibited secretion of TNF-α both at the mRNA and protein levels in overweight mice [106]. Liu et al. have established that metformin (200 mg/kg/14 days) protected the brain from ischemic injury by mitigating inflammatory responses in transient middle cerebral artery occlusion (tMCAO). They also demonstrated that, in mice treated with metformin, neurobehavioral outcomes were considerably improved. Furthermore, they provided evidence that metformin alleviated neutrophil infiltration, hence diminishing endothelial injury and reducing BBB permeability. They also demonstrated that metformin reduced IL-1β, IL-6 and TNF-α expression in tMCAO mice as well as downregulated intercellular adhesion molecule 1 (ICAM)-1 expression via the AMPK signalling pathway [107]. The diminished amount of mRNA transcripts for TNF-α and IL-6 after metformin intake has also been confirmed by Nakatake et al. [108]. They also concluded that metformin treatment inhibited the production of NO and suppressed the mRNA expression of inducible nitric oxide synthase (iNOS) in macrophages, further supporting metformin anti-inflammatory action [108].

There is a strong relationship between metabolic state and immune tolerance. It is associated with immune cells directly exerted by specific intracellular nutrient-energy sensors. Long-term caloric restriction (CR) causes many metabolic and physiologic changes that could be beneficial to MS patients. It has been shown that chronic CR ameliorates the clinical parameters in an EAE mice model [109]. A study demonstrated AMP-activated protein kinase downregulation and enhanced gluconeogenic and lipogenic genes expression observed in the CR rat liver [110]. However, data on the CR effect in the course of MS are missing. Presently, there is an ongoing clinical trial aiming to verify the altering of metabolic state through CR application. The purpose of this trial is to re-equilibrate immune/inflammatory response of RRMS patients and to improve the efficacy of first-line drug treatments [111]. Certainly, CR is very effective, but it seems impractical outside of the research environment. Furthermore, it is difficult to enforce it in long-term human therapy. Therefore, it is preferable to develop a compound that imitates the CR effect without dietary restrictions. The data demonstrated that metformin’s properties allow us to categorize it as a potential candidate to CR mimetic (CRM) group [112]. The results also suggest that metabolism modulation (including glucose and lipid metabolism) and inflammatory response could be major targets in the development of CRMs process [113,114,115]. It should also be mentioned that MS patients are usually incapable of excessive strenuous physical activity due to their motor dysfunctions and neurological disorders. In addition, dietary restrictions can result in exhaustion of a disease-debilitated body. Therefore, CRMs like metformin could be introduced to MS treatment in order to improve metabolic parameters and to potentially inhibit the inflammation and neurodegeneration processes.

## 4. The Effect of Metformin on Haemostasis and the Functioning of Blood Platelets

The immunomodulatory function of the intrinsic coagulation pathway remains unclear in MS pathophysiology. There is a growing interest in a more detailed investigation of the MS coagulation pathway. It will involve cross interactions between inflammation, immunity and neurodegeneration. The key event in MS pathophysiology is BBB disruption, leading to a massive influx of blood elements (nearly all haemostasis factors) into the CNS [116,117]. Except for their role of a possible trigger of the coagulation cascade, haemostasis elements are involved in immune activation, inflammatory response and the process of inducing neurodegenerative sequalae in the MS course. Depending on the BBB injury degree, high-molecular blood components, such as fibrinogen or coagulation factors, can enter into the CNS, thus providing a complete set of factors to trigger pathology coagulation [118]. Fibrinogen is one of the most abundant proteins in human plasma and has been identified as a pivotal contributor to neuroinflammation in MS patients [119,120,121].

Studies have confirmed the beneficial efficacy of metformin on fibrinolysis [122,123,124]. Inhibitors of haemostasis proteases, such as plasminogen activator inhibitor 1 (PAI-1), tissue factor inhibitor (TFPI) and thrombomodulin (TM), are the key regulators of fibrinolysis and coagulation. The PAI-1 level has been reported to be higher in MS patients during exacerbations. The genetic polymorphisms of PAI-1 are linked to lower PAI-1 plasma levels and are associated with increased risk of developing MS syndrome [125,126]. Furthermore, there is a statistically positive correlation between expanded disability status scale (EDSS) scores and TM levels in MS patients [127]. Studies conducted on 138 MS patients (85 RRMS and 53 P-MS) have demonstrated higher PAI-1 and TFPI levels in MS patients compared to healthy individuals [128]. Studies have shown that metformin decreases coagulation factor VII and the circulating level of PAI-1. Furthermore, it interferes with factor XIII, cross-linking activity and fibrin polymerization [129]. Therefore, ex vivo formed clots are easier to lyse in the presence of metformin [130]. In addition, studies have indicated that metformin may decrease the production or the metabolism of tissue-type plasminogen activator (tPA) and von Willebrand factor (vWF), mainly secreted by the endothelial cells [131].

Fibrin clot formation, leading to vessel occlusion, represents the terminal step in the atherothrombotic process and involves a variety of interactions between coagulation factors and blood platelets [132]. Many studies, including our research, indicate an increased number of platelet-derived microparticles and enhanced P-selection expression, which are accepted markers of platelet activation in EAE/MS [133,134,135,136,137,138,139,140,141]. Disruption of the BBB permeability causes a massive infiltration of many leukocytes that elicit neurotrophic changes, leading to demyelination in the CNS [142]. Activation of blood platelets significantly affects interactions between leukocytes and endothelial cells. It is considered that endothelial dysfunction and an increased activation of platelets contribute to the development of neurodegeneration in MS. During the development of inflammation, the activated platelets adhere to the injured endothelium and interact with proteins on the subendothelial layer as well as form platelet-leukocyte complexes near the damaged vessel [143]. Due to the enormous number of surface receptors on activated platelets and biologically active compounds secreted from their granules, these receptors are engaged in the inflammatory process. Diverse interactions between activated platelets and immune cells, and their accumulation in the damaged endothelium promote neurovascular inflammation [144]. In MS, there is an elevated number of platelet granule secreted markers, such as platelet factor-4 (PF-4) and β-thromboglobulin (β-TG). The positive correlation between the plasma level of PF-4 and the acuteness of the MS course was observed [139]. It has been shown that PF-4 reinforces neutrophil adhesion to endothelial cells and degranulation as well as promotes monocyte differentiation into macrophages [145], leading to phagocytosis and generation of reactive oxygen species (ROS) [146]. Furthermore, chemokine ligand (CCL5/RANTES) secreted from platelet granules leads to the promotion of monocyte recruitment to the injured endothelium [147]. Activated platelets produce inflammatory lipid mediators, such as platelet-activating factor (PAF), which is involved in endothelial permeability [148].

The latest research has demonstrated that metformin reduced platelet aggregation by approximately 20%. Moreover, metformin diminished spontaneous platelet adhesion by 27.7% and adenosine diphosphate (ADP)-induced adhesion to fibrinogen by 29.6% compared to the control group [149]. The latest reports relating to cardiovascular disease confirm an enhanced risk of cardiovascular events, such as ischemic stroke, myocardial infarction and thrombosis in MS patients, which are directly associated with coagulation cascade and aberrant platelet function, and their increased pro-thrombotic activity [150,151,152,153,154,155]. What is especially noteworthy is the fact that MS patients do not lead an active lifestyle, which favours the occurrence of ischemic stroke and is primarily related to the high risk of venous thrombosis [156,157,158]. Data obtained by Alzahrani et al. suggest that metformin can ward off both venous and arterial thrombosis via inhibiting platelet activation, especially without significant risk of bleeding. Furthermore, it has been shown that metformin therapy is associated with an increased likelihood of fibrinolytic incidents, which provides additional evidence that metformin has cardioprotective properties [159].

**Figure 1:**
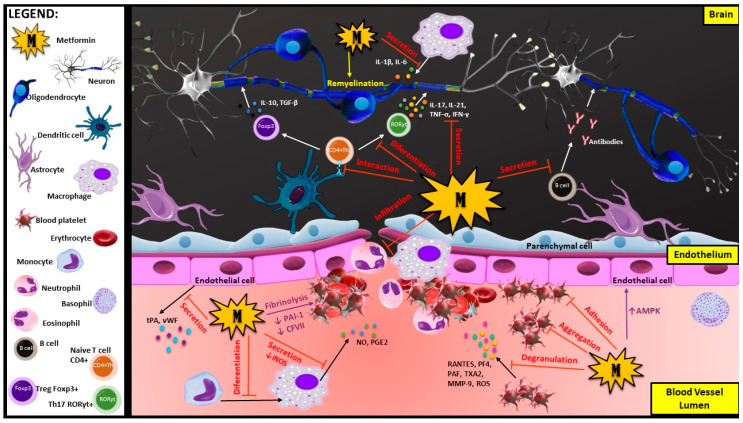
Actions of metformin on immune cells, blood platelets and endothelium during the development of neurodegeneration disease: Metformin has multiple properties such as anti-inflammatory, antioxidant and antithrombotic properties. It may influence different types of cells, including immune cells (oligodendrocytes, macrophages, T cells and B cells), blood platelets and endothelial cells. It may also affect many processes involved in BBB disruption and demyelination during MS development, including pro-inflammatory cell differentiation, degranulation and infiltration of immune cells into the CNS from the blood vessels, as well as myelin repair process (remyelination). As a versatile compound, metformin is responsible for maintaining the Th17/Treg balance by blocking differentiation of CD4+ naive T cell into pro-inflammation RORyt+Th17 cells and stimulation to differentiate into anti-inflammation FoxP3+ Tregs. These actions provide protection against the negative influence of pro-inflammatory cytokines (IL-17, IL-21, TNF-α and IFN-γ) and enhance myelin reconstruction, supporting the remyelination process. Metformin inhibits differentiation of monocytes into macrophages, diminishing the secretion of inflammatory agents (NO, PGE2, IL-1 and IL-6), and inhibits the autoreactive antibodies secretion from B cells. One of the key functions of metformin is blocking the massive infiltration of immune cells (mainly neutrophils and macrophages) into the CNS, which initiates neuroinflammation in MS. This diabetic drug improves the angiogenic functions of endothelial cells by activating the AMPK pathway. Metformin has been shown to stimulate fibrinolysis by decreasing the PAI-1 and CFVII levels in serum as well as has been proven to have an impact on blocking the platelet pro-thrombotic activities by diminishing their adhesion and aggregation. Furthermore, metformin may inhibit the activation of blood platelets, which reduces the secretion of pro-inflammatory cytokines that pull immune cells to the BBB disruption site. Abbreviations: AMPK—adenosine monophosphate kinase; BBB—blood-brain barrier; CFVII—coagulation factor VII; CNS—central nervous system; IFN-γ—interferon γ; IL—interleukin; MMP-9—matrix metalloproteinase 9; MS—multiple sclerosis; NO—nitric oxide; PAF—platelet-activating factor; PAI-1—plasminogen activator inhibitor 1; PF4—platelet factor 4; PGE2—prostaglandin E2; RANTES—regulated upon activation, normal T cell expressed and presumably secreted; ROS—reactive oxygen species; TGF-β—transforming growth factor β; Th17—T helper 17; TNF-α—tumor necrosis factor α; tPA—tissue-type plasminogen activator; TXA2—thromboxane A2; vWF—von Willebrand factor.

Oxidative stress causes cell damage by oxidizing critical cellular components, such as lipids, proteins and DNA, which in consequence may lead to cell death [160]. It is considered that oxidative stress is an essential agent in MS development [161,162,163]. In the acute phase, oxidative stress initiates inflammatory processes, while in the chronic phase, it maintains neurodegeneration [164]. In MS, redox processes are associated with mitochondrial dysfunction, dysregulation of axonal bioenergetics as well as impaired oxidant/antioxidant balance [165,166]. Mitochondrial abnormalities, like mtDNA defects, aberrant mitochondrial gene expression and abnormal mitochondrial enzyme activities, lead to the eventual death of dendritic cells (DCs) and increase the demyelination process [167]. Protti et al. have shown that, in blood platelets from metformin-treated patients, the mitochondrial membrane was hyperpolarized and the activity of complex I was reduced [168]. The above results have been confirmed and enriched with further information the effect of metformin on platelet function through the multiparameter studies conducted by Xin et al. [169]. In the presence of metformin, the pivotal markers of platelet activation, such as αIIbβ3 expression, P-selectin and concentration of cytosolic calcium, were reduced both in vitro and in vivo [169]. They have demonstrated that metformin prevents both venous and arterial thrombosis by inhibiting platelet activation and by realizing extracellular mitochondrial DNA (mtDNA) with no significantly prolonged bleeding time [169]. They discovered three crucial indexes for evaluating the mitochondrial function: mitochondrial membrane potential (MMP), intracellular ATP level and mitochondrial routine respiration in quiescent platelets were improved after metformin treatment. The obtained results demonstrated that metformin protected against mitochondrial dysfunction in blood platelets by inhibiting complex I activity in the respiratory chain and by hyperpolarizing the mitochondrial membrane. They further determined that, through treatment with metformin, activated platelets improved the hyperpolarization level of MMP, intracellular ATP level and mitochondrial functioning [169]. Additionally, mean platelet volume (MPV) was observed to be elevated in patients with high thrombogenic activation. It was shown that the size of platelets, represented by MPV, is strictly related to their reactivity. The sizable platelets are metabolically and enzymatically more active and secrete more inflammatory mediators (i.e., ATP, thromboxane A2 (TXA_2_), serotonin and PF-4), leading to inflammation and atherogenesis [170]. Metformin exhibits an antiatherogenic effect by decreasing MPV and its mass [171]. There are studies showing that metformin reduced MPV in patients with Parkinson’s disease in whom related platelet dysfunctions were confirmed [172]. All the aforementioned actions of metformin are presented in Figure 1 and included in the summary table (Table 1).

## 5. The Effect of Metformin in EAE/MS in Pre- and Clinical Trials

Presently, a vast amount of research on the EAE animals model treated with metformin is conducted, but unfortunately, still little is known about metformin effect on MS patients. A recent cohort study led by Negrotto et al. on metformin-treated MS patients (850–1500 mg/day) demonstrated a significant reduction in the number of new or expanding T2 brain lesions. The effect was observed after 6 months of therapy and lasted up to 24 months. Furthermore, the study has shown an elevated AMPK expression, decreased production of IFN-γ- and IL-17, and increased percentage of Tregs in metformin supplemented MS patients [173]. The latest study conducted by Neumann et al. demonstrated that metformin restored the regenerative capacity of aged OPCs. This study showed that, as rats get older, their OPCs lose the ability to readily mature into functional oligodendrocytes [175]. Aged OPCs become insensitive to differentiating the signals, and this functional capacity deterioration is associated with diminished cellular metabolism and increased susceptibility to DNA damage. The obtained results have shown that metformin promotes remyelination in the stem cells of elderly rats. Aged OPCs treated with metformin regain their sensitivity to pro-differentiation signals. These reports suggest that the application of a biguanidine derivative can re-establish the regenerative capacity of aged OPCs. The ameliorating remyelination in elderly animals following focal demyelination is also observed [175]. The results of the abovementioned studies seem to be particularly promising for considering metformin not only as an anti-inflammatory or antiaging agent but also as a factor in directly influencing remyelination process in MS course. After discovery of the aforementioned phenomenon, the “old drug” has become the subject of newly focused research, and since then, clinical trials on MS patients have been appearing. Presently, there is an ongoing clinical trial (phase I double-blind study) investigating the metformin effect on endogenous neural progenitor cells in MS children (the completion is expected in October 2022). The subjects were randomized into two double-blinded groups receiving either metformin (2 × 850 mg per day) or a placebo in combination with moderate energy restriction and exercise regimens (30 min per day for 1 year) [176]. There is also a clinical trial planned at the University of Cambridge in which the metformin in combination with clemastine as potential remyelination therapy will be tested [177].

## 6. Conclusions

Metformin is a versatile drug with a long history and rich prospects for the future. The main effect of metformin is the reduction of insulin resistance, but new functions are constantly being discovered. Metformin shows desirable properties in EAE/MS therapy, including antioxidant, anti-inflammatory or antiplatelet function. Unfortunately, there are very few clinical studies conducted on patients with MS that could unequivocally confirm the effective impact of metformin. Nowadays, the potentially beneficial effect was observed on the EAE animal model, which does not always correspond with human disease pathophysiology. Considering the similar metformin molecular mechanisms and some approved compounds, a beneficial biological metformin effect in MS therapy is expected. More clinical trials to analyse the metformin effect on disability parameters, the level of inflammatory markers and quantification of neuronal damage in MS patients are required.

## Figures and Tables

**Table 1:** Documented anti-inflammatory effects of metformin application in MS/experimental encephalomyelitis (EAE) and potential antithrombotic/antioxidant effects.

Anti-Inflammatory Effect	Metformin Dose	MS/EAE	Ref.
-slows disease progression -reduces the level of inflammatory cells in CNS -decreases expression of pro-inflammatory cytokines (IL-1β, IL-6, IL-17, IFN-γ and TNF-α) in the CNS -inhibits the expression of MMP-9 and RANTES	20–100 mg/kg/day	EAE (C57BL/6 and SJL mice)	[101]
-reduces the number of new and expanding T2 lesions in brain -decreases the production of IFN-γ and IL-17 and elevates the level of AMPK expression in PBMCs -enlarges Tregs percentage	850–1500 mg/kg/day	RRMS	[173]
-reduces the Th17 percentages, and promotes Tregs proliferation -enhances the levels of anti-inflammatory cytokines transforming growth factor-β (TGF-β) and IL-10 -suppresses activation of mammalian target of rapamycin (mTOR) pathway	100 mg/kg/day	EAE (C57BL/6 mice)	[99]
-attenuates the clinical symptoms in established EAE animals via inhibition of cellular infiltration and T cell autoimmunity -enhances the level of Tregs and reduces the level of Th1 and Th17 in peripheral nervous system (PNS) and CNS compartments -reduces the demyelination and axonal loss	150 mg/kg/day	EAE (Lewis rats)	[74]
-attenuates inflammatory response, and enhances expressions of neurotrophic factors, hence protecting oligodendrocytes via AMPK activation -attenuates oxidative stress level	150 mg/kg/day	EAE (Lewis rats)	[78]
Potential Antithrombotic and Antioxidant Effects of Metformin		Ref.	
-reduces the platelet aggregation and adhesion		[149]	
-decreases the GPIIb/IIIa and P-selectin expression on platelets -diminishes the intracellular level of calcium in platelets -reduces the activity of complex I in the respiratory chain in platelets mitochondria, and diminishes hyperpolarization of the mitochondrial membrane in platelets -inhibits mtDNA release from mitochondria present in platelets -reduces ROS production and ROS-induced membrane damage		[169]	
-enhances the fibrinolysis process		[159]	
-reduces the level of vWF and tPA form endothelial cells -restores endothelial dysfunction		[131]	
-decreases secretion of CFVII and PAI-1 in plasma		[174]	
